# Gastric Xanthelasma, Microsatellite Instability and Methylation of Tumor Suppressor Genes in the Gastric Mucosa: Correlation and Comparison as a Predictive Marker for the Development of Synchronous/Metachronous Gastric Cancer

**DOI:** 10.3390/jcm11010009

**Published:** 2021-12-21

**Authors:** Masashi Fukushima, Hirokazu Fukui, Jiro Watari, Chiyomi Ito, Ken Hara, Hirotsugu Eda, Toshihiko Tomita, Tadayuki Oshima, Hiroto Miwa

**Affiliations:** Division of Gastroenterology and Hepatology, Department of Internal Medicine, Hyogo College of Medicine, Nishinomiya 663-8501, Japan; ma-fukushima@hyo-med.ac.jp (M.F.); watarij@kinentou.or.jp (J.W.); s.aurantiaca66@gmail.com (C.I.); k-hara@hyo-med.ac.jp (K.H.); eda@hyo-med.ac.jp (H.E.); tomita@hyo-med.ac.jp (T.T.); t-oshima@hyo-med.ac.jp (T.O.); miwahgi@hyo-med.ac.jp (H.M.)

**Keywords:** gastric xanthelasma, synchronous/metachronous gastric cancer, endoscopic submucosal dissection, genetic alteration, predicting marker

## Abstract

A predictive marker for the development of synchronous/metachronous gastric cancer (GC) would be highly desirable in order to establish an effective strategy for endoscopic surveillance. Herein, we examine the significance of gastric xanthelasma (GX) and molecular abnormalities for the prediction of synchronous/metachronous GC. Patients (*n* = 115) were followed up (range, 12–122; median, 55 months) in whom the presence of GX and molecular alterations, including microsatellite instability (MSI) and methylation of *human mutL homolog 1* (*hMLH1*), *cyclin-dependent kinase inhibitor 2A* (*CDKN2A*) and *adenomatous polyposis coli* (*APC*) genes, had been confirmed in non-neoplastic gastric mucosa when undergoing endoscopic submucosal dissection (ESD) for early GC. At the start of surveillance, the numbers of positive subjects were as follows: GX, 59 (51.3%); MSI, 48 (41.7%); *hMLH1*, 37 (32.2%); *CDKN2A*, 7 (6.1%); *APC*, 18 (15.7%). After ESD treatment, synchronous/metachronous GCs occurred in patients with the following positive factors: GX, 16 (27.1%); MSI, 7 (14.6%); *hMLH1*, 6 (16.2%); *CDKN2A*, 3 (42.9%); *APC*, 3 (16.7%). The presence of GX had no significant relationship to positivity for MSI or methylation of *hMLH1*, *CDKN2A* or *APC*. GX was significantly (*p* = 0.0059) and independently (hazard ratio, 3.275; 95% confidence interval, 1.134–9.346) predictive for the development of synchronous/metachronous GC, whereas those genetic alterations were not predictive. GX is a simple and powerful marker for predicting the development of synchronous or metachronous GC.

## 1. Introduction

Gastric cancer (GC) is still a leading cause of cancer-related mortality, especially in eastern countries [1,2]. With advances in endoscopy and diagnostic strategies, a considerable number of GCs can now be detected at an early stage and be treated curatively by endoscopic submucosal dissection (ESD) [3,4]. However, after endoscopic treatment of GC, the development of synchronous and/or metachronous lesions is a concern during follow-up [5,6]. For this reason, although endoscopic surveillance is recommended, no specific strategies for assisting the frequency or risk of such lesions have yet been established. Therefore, to improve the efficiency and effectiveness of endoscopic surveillance, a predictive marker for the development of synchronous/metachronous GC would be highly desirable.

It has been accepted that irreversible accumulation of molecular abnormality occurs in precancerous conditions, i.e., atrophic gastritis and intestinal metaplasia with chronic *Helicobacter pylori* (*H. pylori*) infection [7]. Thus, the whole of the gastric mucosa with *H. pylori* infection has a high potential for development of GC, which is consistent with the frequent occurrence of synchronous/metachronous GC in this situation. Interestingly, it has been shown that microsatellite instability (MSI) or methylation of tumor-suppressor genes frequently occurs in the non-neoplastic gastric mucosa of patients with GC [8,9,10] and that, moreover, these molecular abnormalities do not completely normalize, even after successful eradication of *H. pylori* [7,8,11]. These findings offer a good explanation of the relatively high frequency of GC development, even after successful eradication of *H. pylori* infection. In these contexts, molecular abnormalities in the gastric mucosa may be candidate markers for prediction of the development of GC [10,12,13]. We recently reported that the incidence of molecular events related to carcinogenesis was mostly observed in IM, with very few in atrophic mucosa without intestinal metaplasia [14]. On the other hand, we recently reported that gastric xanthelasma (GX), characterized by accumulation of lipid in histiocytic foam cells [15], is a useful marker for prediction of the development of GC [16,17]. In the present study, therefore, we analyzed the correlation between GX and molecular abnormalities in the gastric mucosa, especially in intestinal metaplasia, of patients with early GC and investigated its significance for prediction of synchronous/metachronous GC.

## 2. Materials and Methods

### 2.1. Patients

This was a cohort study following our previous investigation in molecular alterations in the non-neoplastic gastric mucosa of patients with early GC [8]. Written informed consent had been obtained from all patients involved in the previous study (Ethics Nos. 136 and 154), and the opt-out for this observational study (Ethics No. 0404) was announced on the website of Hyogo College of Medicine. All studies were approved by the Ethics Committee of Hyogo College of Medicine.

A total of 115 patients were investigated in this study. All patients satisfied the following criteria: 1, subjects who had undergone ESD for GCs between August 2010 and December 2013; 2, subjects who had been enrolled in the previous study [8] and examined for molecular alterations in non-neoplastic gastric mucosa when receiving ESD treatment; 3, subjects who had been followed-up for ≥12 months by endoscopy to examine whether synchronous or metachronous GC had occurred after ESD treatment.

In the present study, we used the criteria of the Japanese Research Society for Gastric Cancer as the histological criteria for gastric cancer. The criteria of Kimura and Takemoto, reported previously [18,19], were also adopted for the severity of gastric atrophy. Endoscopists (M.F., K.H. and H.E.), who were blinded to the data of molecular alterations and patients’ clinical course, confirmed the presence of xanthomas in endoscopic examination before ESD treatment, retrospectively. At the time of ESD, the status of *H. pylori* infection was determined by Giemsa staining of gastric biopsy samples and the obtained serum level of anti-*H. pylori* antibody and then defined as positive if at least one test gave a positive result. If *H. pylori* had been eradicated after ESD, the status of infection was examined by urease breath test.

### 2.2. Analyses of MSI and Gene Methylation

Molecular alterations in the intestinal metaplasia were analyzed as described previously [8]. In brief, biopsy specimens of non-neoplastic gastric mucosa at the greater curvatures of the antrum and corpus and the lesser curvature of the angulus were embedded in paraffin blocks. Seven-micrometer-thick tissue sections were cut, samples of epithelial cells were isolated by laser microdissection, and DNA was extracted only from the goblet intestinal metaplasia glands (incomplete type) using a QIAamp DNA Micro Kit (Qiagen, Hilden, Germany).

We examined five microsatellite loci on chromosomes for MSI based on the revised Bethesda panel [20], as follows: 2p (BAT26), 4q (BAT25), 2p (D2S123), 5q (D5S346) and 17p (D17S250). The MSI status was judged as previously reported [8]. To analyze the genetic methylation status, extracted DNA was modified using sodium bisulfite with the EpiTect Plus DNA Bisulphite Kit (Qiagen, Hilden, Germany). The modified DNA was amplified using specific primer pairs for the methylated or unmethylated sequences of *human mutL homolog 1* (*hMLH1*), *cyclin-dependent kinase inhibitor 2A* (*CDKN2A*) and *adenomatous polyposis coli* (*APC*) [8]. Thereafter, the methylation status of those genes was examined by methylation-sensitive high-resolution melting analysis, as previously described [8,21]. A methylation standard curve was prepared using a set of methylated (100%) and unmethylated (0%) DNA (EpiTect PCR Control DNA Set; Qiagen, Hilden, Germany). The methylation status of each target gene was scored as low (<10%), moderate (≥10% to <50%) or high (≥50%). Samples with a moderate or high methylation level were considered to be methylated.

### 2.3. Statistical Analysis

The Statview 5.0J statistical software package (Abacus Concepts Inc., Berkeley, CA, USA) was used for all analyses in the present study. Data for age and BMI were expressed as the mean ± SD, and categorical data were presented as frequencies with proportion. Differences in age and BMI between two groups were analyzed by unpaired two-tailed *t* test or by Mann-Whitney *U*-test when the data were not parametric. Fisher’s exact test was performed to investigate the relationships between groups and clinical/genetic features. Cumulative incidence of synchronous/metachronous GC development after ESD treatment was evaluated by the Kaplan-Meier method and analyzed by log-rank test. Differences at *p* < 0.05 were considered to be statistically significant.

## 3. Results

### 3.1. Relationship of GX to Clinical/Endoscopic Features in Patients with Early GC Treated by ESD

Table 1 summarizes the clinical and endoscopic features of patients with early GC treated by ESD (*n* = 115). Most of the patients (*n* = 108, 93.9%) had open-type gastric atrophy. Eighty-nine patients (77.4%) were positive for *H. pylori* infection, and 11 were negative after *H. pylori* eradication. Fifteen were negative for *H. pylori* infection without eradication, and 13 of them had atrophy, suggesting previous *H. pylori* infection.

GX was detected in 59 (51.3%) of the 115 patients investigated. None of the parameters—age, sex, BMI, severity of gastric atrophy or *H. pylori* infection status—showed a significant relationship to the prevalence of GX.

### 3.2. Relationship of MSI or Methylation of hMLH1, CDKN2A or APC to Clinical/Endoscopic Features in Patients with Early GC Treated by ESD

Among 115 patients with early GC who underwent ESD, 48 (41.7%) were positive for MSI (Table 2). None of the examined parameters—age, sex, BMI, severity of gastric atrophy or *H. pylori* infection status—showed a significant relationship with MSI positivity.

Methylation of the *hMLH1* gene was detected in 37 (32.2%) of the patients with early GC who underwent ESD. Positivity for *hMLH1* methylation showed no relationship with any of the above clinical/endoscopic features either.

Methylation of the *CDKN2A* and *APC* gene was detected in 7 (6.1%) and 18 (15.7%) of patients with early GC who underwent ESD, respectively. Positivity for *CDKN2A* or *APC* methylation showed no relationship with any of the above clinical/endoscopic features either.

### 3.3. Relationship between GX and MSI or Methylation of hMLH1, CDKN2A or APC in Patients with Early GC Treated by ESD

We next investigated the relationship between the prevalence of GX and molecular alterations in the gastric mucosa of patients with early GC (Table 3). Contrary to expectation, we found no significant correlation between the prevalence of GX and molecular alterations of MSI or methylation of *hMLH1*, *CDKN2A* or *APC*.

### 3.4. Significance of GX, MSI and Methylation of Tumor Suppressor Genes as a Predictive Marker for the Development of Synchronous/Metachronous GC

During the follow-up period, synchronous/metachronous GC was found in 21 (18.3%; 5 synchronous and 16 metachronous, respectively) of the 115 patients (Table 4). When investigating according to the prevalence of GX, 16 (27.1%) of the 59 patients with GX developed synchronous/metachronous GC after ESD treatment. On the other hand, 5 (8.9%) of 56 patients without GX had such lesions. As for the prevalence of MSI, 7 (14.6%) of 48 patients with MSI had synchronous/metachronous GC, and 14 (20.9%) of 67 patients without MSI had such lesions. In addition, 6 (16.2%) of 37 patients with *hMLH1* methylation had synchronous/metachronous GCs and 15 (19.2%) of 78 patients without *hMLH1* methylation had such lesions. Three (42.9%) of seven patients with *CDKN2A* methylation had synchronous/metachronous GCs, and 3 (16.7%) of 18 patients with *APC* methylation had such lesions.

**Figure 1:**
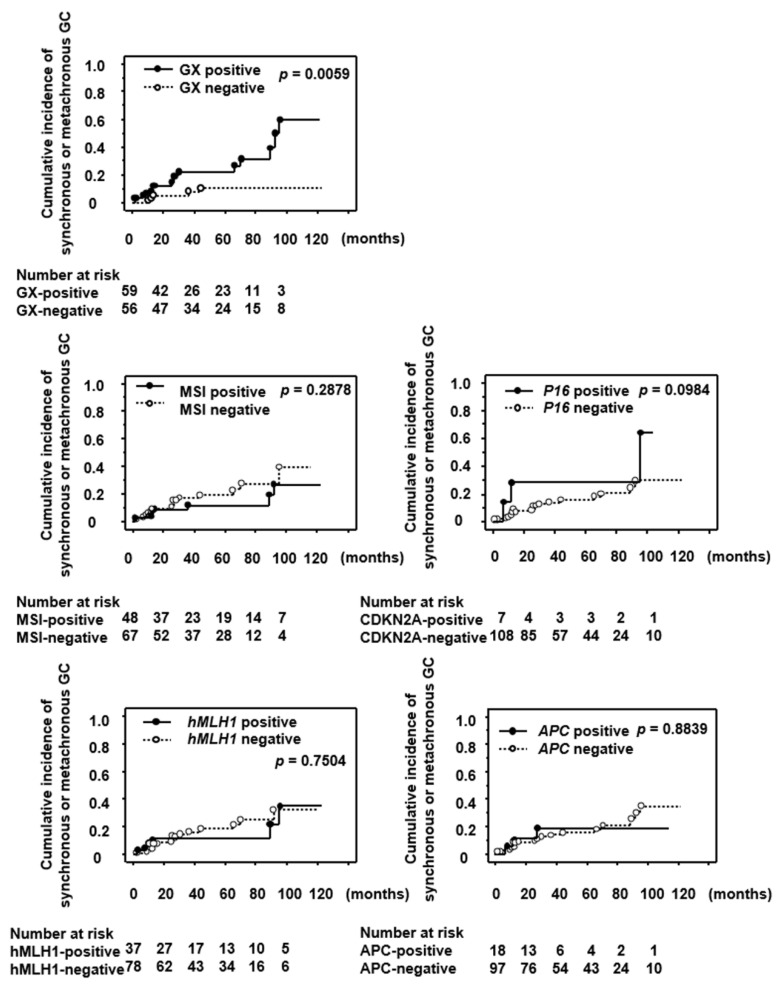
Cumulative incidence of synchronous/metachronous gastric cancer during endoscopic follow-up (median, 55 months; range, 12–122 months) in patients after ESD treatment. GX, gastric xanthelasma; MSI, microsatellite instability; *hMLH1*, *human mutL homolog 1*; *CDKN2A*, *cyclin-dependent kinase inhibitor 2A*; *APC*, *adenomatous polyposis coli*.

Furthermore, we compared the cumulative incidence of synchronous/metachronous GC between GX-positive and -negative cases (Figure 1). Kaplan–Meir curves show that significantly more patients with GX developed synchronous/metachronous GC than those without GX (Figure 1). In terms of the status of MSI and methylation of *hMLH1*, *CDKN2A*, or *APC*, the Kaplan–Meir curves show no significant differences between the groups positive and negative for those genetic alterations (Figure 1).

We next examined whether the presence of GX is an independent factor predictive of synchronous/metachronous GC development. Univariate analysis showed that GX was significantly related to the development of synchronous/metachronous GC (Table 4). Moreover, multivariate analysis clarified that the presence of GX was independently related to the development of synchronous/metachronous GC (Table 4).

## 4. Discussion

On the basis of clinical/endoscopic features, the identification of a predictive marker for the development of synchronous/metachronous GC has long been desirable. Accumulating evidence has revealed that male sex and severe atrophy are independent risk factors for not only initial but also synchronous/metachronous GC [5,6]. In addition, we previously reported that GX is a powerful marker for prediction of the development of GC [16,17]. Moreover, in the present study, we have clarified that GX is a possible marker for prediction of the development of synchronous/metachronous GC, which is consistent with a report by Shibukawa et al. [22]. GX is characterized by accumulation of foamy histiocytes in the inflamed gastric mucosa and is thought to be the result of an inflammatory response to mucosal damage or aging [15,23]. In this regard, one might argue that gastric xanthelasma merely reflects the severity and long duration of gastric atrophy, which is a crucial risk factor for GC development. However, our previous multivariate analysis clearly indicates that GX is a factor independent of gastric atrophy for prediction of the development of GC [17]. Moreover, the present study similarly clarifies its significance as an independent predictor for synchronous/metachronous GC. It has been reported that increased release of oxygen free radicals, which cause DNA damage and play a role in the pathophysiology of various malignancies [24,25], is involved in the formation of GX [15]. Thus, it is tempting to speculate that the presence of GX may reflect the activation of oxygen free radicals and the associated promotion of genetic alterations in the gastric mucosa. In this context, we therefore investigated the relationship between the presence of GX and molecular alterations in the gastric mucosa of patients with GC.

MSI is a form of genetic instability characterized by alterations in the length of the tandem repeat sequence (termed “microsatellite”) [26], owing to inactivation of mismatch repair genes, such as *hMSH2* and *hMLH1* [27], and it is evident that MSI and/or methylation of *hMLH1* is frequent in various malignancies [28]. In addition, the methylation of tumor suppressor *CDKN2A* and *APC* is widely involved in gastrointestinal carcinogenesis by affecting cell cycle or proliferation [29,30,31,32]. In these contexts, we and others have shown that MSI and/or methylation of tumor-suppressor genes, including *hMLH1*, frequently occurs in the non-neoplastic gastric mucosa of patients with early GC [8,10,33] and that these molecular alterations can be potential markers for prediction of the development of GC [10,34]. In the present study, methylation of *CDKN2A* and *APC* was not very frequent in the non-neoplastic gastric mucosa, especially in intestinal metaplasia of patients with early GC and not predictive of the development of synchronous/metachronous GC, suggesting that those gene alterations may not be very critical in gastric carcinogenesis. On the other hand, it is noteworthy that *H. pylori* eradication is unable to normalize any molecular abnormality for MSI and *hMLH1* in patients with early GC who undergo ESD [8], which supports the contention that *H. pylori* eradication cannot necessarily prevent the development of metachronous GC [35]. In this context, it is interesting that GX persists even after *H. pylori* eradication [36] and that its presence is a predictive marker for the development of synchronous/metachronous GC. We then investigated the relationship between GX and the status of MSI or *hMLH1* methylation, but contrary to expectation, no significant correlations were evident. Besides these molecular alterations, considerable patterns of genetic abnormality are involved in the development of GC [37,38,39]. Therefore, it may be an interesting theme to identify the molecular alteration responsible for the occurrence of GX in the gastric mucosa.

We next investigated whether MSI or *hMLH1* methylation in the intestinal metaplasia is predictive for the development of synchronous/metachronous GC, as such molecular alterations may be applicable to prediction of the initial development of GC [10]. However, the results suggest that neither MSI nor *hMLH1* methylation is likely to predict the development of synchronous/metachronous GC in patients after ESD treatment. These results may be reasonable, as several studies have shown that MSI and/or *hMLH1* methylation is not useful for prediction of the development of GC [9,40]. On the other hand, genetic researchers have continuously investigated and identified some candidate molecular markers (methylation of *microRNA-34b/c* and *-124a3* or somatic mutation of *ARID1A* and *MAGI1*) for prediction of the development of metachronous GC [41,42,43]. However, since molecular alterations in GC patients are very complex and diverse [37,38,39], it might be difficult to select a specific genetic marker that can predict the development of synchronous/metachronous GCs.

In summary, although the molecular alteration responsible for the occurrence of GX in the gastric mucosa remains unclear, GX is a powerful marker for prediction of the development of synchronous/metachronous GC, at least compared with molecular alterations of MSI or methylation of *hMLH1*, *CDKN2A* or *APC* in patients with early GC. GX is very easy to detect in routine endoscopic examinations, whereas detection of molecular abnormality needs advanced equipment and technology. Thus, in clinical practice, GX may be a very useful marker for identification of patients, during follow-up surveillance, who are at high risk for development of synchronous/metachronous GC. The possibility that a powerful molecular marker might become available in the future for prediction of the development of synchronous/metachronous GC cannot be excluded. However, we believe that GX is a simple yet very effective marker in patients undergoing endoscopic surveillance for development of synchronous/metachronous GC and that the usefulness of GX should be validated in a large-scale, prospective, multi-center study.

## Figures and Tables

**Table 1:** Comparison of clinical features between patients with and without gastric xanthelasma.

Characteristics	Total Patients (*n* = 115)	Patients with GX (*n* = 59)	Patients without GX (*n* = 56)	*p* Value
**Age**				
≥65	97 (84.3)	52 (88.1)	45 (80.4)	0.3088
<65	18 (15.7)	7 (11.9)	11 (19.6)	
**Sex**				
Male	82 (71.3)	45 (76.3)	37 (66.1)	0.3027
Female	33 (28.7)	14 (23.7)	19 (33.9)	
**BMI**	23.0 ± 3.4	22.9 ± 3.0	23.1 ± 3.9	0.8843
**Atrophy**				
Open-type	108 (93.9)	57 (96.6)	51 (91.1)	0.2928
Closed-type	5 (4.4)	2 (3.4)	3 (5.3)	
None	2 (1.7)	0 (0.0)	2 (3.6)	
***H. pylori***				
Negative	15 (13.0)	5 (8.5)	10 (17.9)	0.2553
Era-negative	11 (9.6)	7 (11.9)	4 (7.1)	
Positive	89 (77.4)	47 (79.6)	42 (75.0)	

GX, gastric xanthelasma; BMI, body mass index.

**Table 2:** Comparison of clinical features between patients with and without MSI or methylation of tumor suppressor genes.

Characteristics	MSI (+) (*n* = 48)	MSI (−) (*n* = 67)	*p* Value	*hMLH1* (+) (*n* = 37)	*hMLH1* (−) (*n* = 78)	*p* Value	*CDKN2A* (+) (*n* = 7)	*CDKN2A* (−) (*n* = 108)	*p* Value	*APC* (+) (*n* = 18)	*APC* (−) (*n* = 97)	*p* Value
**Age**												
≥65	40 (83.3)	57 (85.1)	0.8008	28 (75.7)	69 (88.5)	0.1003	5 (71.4)	92 (85.2)	0.3008	13 (72.2)	84 (86.6)	0.1553
<65	8 (16.7)	10 (14.9)		9 (24.3)	9 (11.5)		2 (28.6)	16 (14.8)		5 (27.8)	13 (13.4)	
**Sex**												
Male	35 (72.9)	47 (70.1)	0.8356	30 (81.1)	52 (66.7)	0.1272	4 (57.1)	78 (72.2)	0.4074	16 (88.9)	66 (68.0)	0.0915
Female	13 (27.1)	20 (29.9)		7 (18.9)	26 (33.3)		3 (42.9)	30 (27.8)		2 (11.1)	31 (32.0)	
**BMI**	23.5 ± 3.6	22.6 ± 3.2	0.3127	22.9 ± 3.0	23.0 ± 3.6	0.8131	21.4 ± 2.3	23.1 ± 3.5	0.1226	21.9 ± 2.8	23.1 ± 3.5	0.2124
**Atrophy**												
Open-type	44 (91.7)	64 (95.5)	0.6750	35 (94.6)	73 (93.6)	0.7285	6 (85.7)	102 (94.5)	0.0284	16 (88.9)	92 (94.9)	0.3836
Closed-type	3 (6.2)	2 (3.0)		1 (2.7)	4 (5.1)		0 (0.0)	5 (4.6)		1 (5.55)	4 (4.1)	
None	1 (2.1)	1 (1.5)		1 (2.7)	1 (1.3)		1 (14.3)	1 (0.9)		1 (5.55)	1 (1.0)	
***H. pylori***												
Negative	4 (8.3)	11 (16.4)	0.3368	4 (10.8)	11 (14.1)	0.8591	1 (14.3)	14 (12.9)	0.8973	1 (5.6)	14 (14.4)	0.5863
Era-negative	6 (12.5)	5 (7.5)		4 (10.8)	7 (9.0)		1 (14.3)	10 (9.3)		2 (11.1)	9 (9.3)	
Positive	38 (79.2)	51 (76.1)		29 (78.4)	60 (76.9)		5 (71.4)	84 (77.8)		15 (83.3)	74 (76.3)	

MSI, microsatellite instability; *hMLH1*, *human mutL homolog 1*; *CDKN2A*, *cyclin-dependent kinase inhibitor 2A*; *APC*, *adenomatous polyposis coli*; BMI, body mass index.

**Table 3:** Relationship between gastric xanthelasma and genetic alterations in early gastric cancer patients.

Characteristics	Patients with GX (*n* = 59)	Patients without GX (*n* = 56)	*p* Value
**MSI**			
positive	23 (39.0)	25 (44.6)	0.5744
negative	36 (61.0)	31 (55.4)	
***hMLH1* methylation**			
positive	19 (32.2)	18 (32.1)	0.9945
negative	40 (67.8)	38 (67.9)	
***CDKN2A* methylation**			
positive	3 (5.1)	4 (7.1)	0.7121
negative	56 (94.9)	52 (92.9)	
***APC* methylation**			
positive	7 (11.9)	11 (19.6)	0.3088
negative	52 (88.1)	45 (80.4)	

GX, gastric xanthelasma; MSI, microsatellite instability; *hMLH1*, *human mutL homolog 1*; *CDKN2A*, *cyclin-dependent kinase inhibitor 2A*; *APC*, *adenomatous polyposis coli*.

**Table 4:** Univariate and multivariate analyses of the cumulative incidence of synchronous or metachronous gastric cancer during endoscopic follow-up in patients after ESD treatment.

Characteristics	Total with Synch or Metach GC/Total Patients	Univariate	Multivariate	
		*p* Value	95% CI	*p* Value
**Age**				
≥65	18/97	0.847	1.096 (0.295–4.074)	0.892
<65	3/18		1.0	
**Sex**				
Male	16/82	0.790	1.828 (0.507–6.579)	0.357
Female	5/33		1.0	
**GX**				
Present	16/59	**0.015**	3.257 (1.134–9.346)	**0.028**
Absent	5/56		1.0	
**MSI**				
positive	7/48	0.468	0.711 (0.273–1.855)	0.486
Negative	14/67		1.0	
***hMLH1* methylation**				
Positive	6/37	0.800	0.512 (0.142–1.842)	0.305
Negative	15/78		1.0	
***CDKN2A*** **methylation**				
Positive	3/7	0.113	4.673 (0.671–32.258)	0.120
Negative	18/108		1.0	
***APC* methylation**				
Positive	3/18	0.849	1.300 (0.335–5.051)	0.705
Negative	18/97		1.0	

GX, gastric xanthelasma; MSI, microsatellite instability; *hMLH1*, *human mutL homolog 1*; *CDKN2A*, *cyclin-dependent kinase inhibitor 2A*; *APC*, *adenomatous polyposis coli*.

## Data Availability

Any data referred to in this work will be available on request.
